# International Dental Federation: First General Meeting, Held at Cambridge, England, 1901

**Published:** 1901-11

**Authors:** 


### INTERNATIONAL DENTAL FEDERATION: FIRST GENERAL MEETING, HELD AT CAMBRIDGE, ENGLAND, 1901.

The first meeting of the Federation was held in the Physiological Theatre, University Museums, on the morning of Wednesday, August 7, when the Federation was welcomed to Cambridge by the deputy vice-chancellor of the university, Sir Michael Foster, M.D., F.R.S., M.P., who said:

Mr. President and Gentlemen,—The vice-chancellor of the University of Cambridge is, unhappily for us, obliged to be away from the university at this period, and in his absence he has asked me to act as his deputy and to bid a most hearty welcome to this important International Dental Federation. I understand that its international character is assured by the participation in it of seventeen different countries, and I assure you that this ancient town feels it a compliment that you have chosen it as one, if not the very first, for your visit. The vice-chancellor trusts that your visit here will be both profitable and agreeable: that it will be profitable will rest mainly with yourselves; that it shall be agreeable we have done our best to insure.

*The President* (Dr. Godon).—Sir Michael Foster, ladies and gentlemen, permit me, in the name of my colleagues of the International Dental Federation, to thank the vice-chancellor and the members of the council of the University of Cambridge for the kind hospitality that has been tendered us in these ancient buildings, where generations of students and professors, many of whom have become illustrious, have succeeded one another. No place could be more appropriate for our labors than Trinity College, where the names of Newton, Roger Bacon, Macaulay, Tennyson, Dryden, and many others present themselves spontaneously to our minds to inspire us and to encourage us in the work of universal union and of international education that we have undertaken.

And no one is better qualified to receive us than Sir Michael Foster, the learned representative of the vice-chancellor of the university; Sir Michael Foster, the eminent physiologist whose name has become universally famous through his scientific work. He welcomes us to-day with the same kindness with which, as president of the British Association, he welcomed my countrymen at Dover in 1899. In the name of the Executive Council of the International Dental Federation, and in that of the International Commission of Education, I beg to tender him the sincere expression of our gratitude and respect.

*Sir Michael Foster.*—Mr. President and gentlemen, the ancient university to which I have just had the pleasure of bidding you welcome, and which numbers among its illustrious men, in addition to the names which your president has mentioned, that of William Harvey, presents somewhat mediæval features which are lost to other universities,—features mediæval, but modified by modern development.

In the earliest days of the university every one who attained the title of Doctor thereby gained the right to teach. He, in those early days, taught in any room he could, in one which he hired for the purpose with his own scanty earnings, or in one which was granted to him by the benevolence of others. His pupils in like manner lived where they could, sometimes in such lodgings as their poor purse could secure, sometimes enjoying the hospitality of benefactors. In the course of time the university became able to make provision for its teachers,—if not for all its doctors, at least for those whom after a while it came to speak of as professors. The students, on the other hand, found it to their profit to gather together in common lodgings, which came to be called hostels.

In most countries other than England, while the provision made by the university for its teachers has enjoyed a large development and all universities have now their lecture theatre, their museums, their libraries, their laboratories, and their halls for solemn occasions, the hostels have for the most part been broken up and the students left to shift for themselves. In England, on the other hand, the country having been for centuries secure on the whole from invasion and war's destructive effects, the hostels have flourished more and more. In course of time, after in some instances a temporary connection with religious orders, they have developed into what we here call colleges,—institutions which are hostels in the sense that they afford lodgings for the students, but which do much more than this, in that, over and above what is done by the university, they afford teaching of a very varied kind, and moreover have entered into special relations with the university itself. Each college, in fact, is in many respects, in Cambridge, a small university within the mother one. Here at Cambridge we have seventeen colleges, in addition to institutions which we consider as and call mere hostels, seventeen small universities having complicated relations with the university itself and carrying out much of the teaching,—performing, in fact, almost all university functions save that of giving a degree.

Such a state of things could not help leading to a certain rivalry between the mother and the seventeen daughters. The prosperity of the colleges was more or less inimical to that of the university, and indeed for many years the university, as distinct from the colleges, somewhat languished. During the last generation or so, however, it has undergone great development and expansion.

You are gathered to-day in a university which, like its sister university of Oxford, bears more distinctly than do most of the other universities of Europe the stamp of early and mediæval times, preserved by the predominance of the colleges. You may recognize this in the direction and respective relations of the studies carried on in the plan. In old times there were three faculties in a university,—Theology, Law, and Medicine, corresponding to the three pursuits which demanded at that time book-learning. For the university was founded for practical purposes, and only these three pursuits as yet needed book-learning; the soldier, the merchant, and the manufacturer could do without it. Later on there grew up a faculty of Arts for the protection and advancement of those more general studies which furnished an introduction to the three special practical studies. He who aspired to be a doctor of theology, law, or medicine spent much time in this common learning before he specialized for his profession. In the course of time the colleges took up with vigor this common learning, leaving the more professional studies to the university itself. Moreover, partly from the circumstances of their origin, their early connection with religious orders, partly from other influences, the colleges, and with the colleges the university, became more and more associated with the church, the Established Church of England. And, indeed, during the early and even the middle part of the past century the university and the colleges seemed to belong to the church. The university became the training-place for nearly all the clergy, and gave them all they needed, while some lawyers only, and even fewer doctors, sought its aid, and received there not a professional, but solely a general education. The last generation has, however, seen great changes. The ties with the church have been loosened, professional studies have been encouraged, and in an increasing manner not clergymen, lawyers, and doctors only, but men of other professions and pursuits,—the engineer, the farmer, the man of business and commerce, the manufacturer, and even the soldier,—are knocking at its doors and seeking for professional as well as general education.

At the present moment you will find this university, like other seats of learning and education, busy with the question, What is the best kind of education for each profession and pursuit?—a question which is also stirring you.

All, I venture to think, are agreed that education should be fashioned after the manner of a cone, starting from a broad basis and narrowing to an apex, for it is the conical bullet that has penetrating powers. In the storm and stress of modern life an all-round education, such as makes a man a mental sphere, is not in itself adequate. Spheres move readily one over the other, and spherical education may be good in society, but it is not suited for a profession. The round ball thrown at a surface may make a hole, but more frequently simply rebounds; whereas the cone may be depended upon to pierce, and the man whose education is conical makes his way.

For each profession the cone should be different, should be fashioned in different ways, though in each case it should start from the same broad basis,—namely, the broad basis of the discipline of the school; that is, the boy's school. I say discipline rather than the learning of the school, for the aim of the school master should be in all cases the formation of the mind,—the setting of the instrument, not the filling of the bottle. The growth of habits of accuracy, of intentness, and of alertness, this rather than the gathering of mere knowledge of facts, is the proper heritage of the school, and for the attainment of these habits it matters not so much what the boy is taught as how he is taught.

From this broad basis of a general school education the narrowing of professional training begins, and we thus come to the question which interests us to-day; that is, the narrowing of training which is best for the dentist, and how shall it best be brought about? On this it would not be fitting that I should do more than offer a few general reflections.

The dentist is a healer; his business lies with a very small portion of the human frame, but that portion, though small, is still human; it has its diseases, its failings, and the dentist has to cure these, bringing in, whenever it be possible, that best of cures, prevention. The training of the dentist is, in broad terms, the training of a healer.

I remember that in my young days a celebrated surgeon used to say that a surgeon was a physician and something more, meaning that he had to possess a general knowledge of disease such as the physician possesses, but had, in addition, not only to know certain features of disease which the physicians might neglect, but also to acquire a manual dexterity which the physician never needed. In somewhat the same way we may say that the dentist is a surgeon and something more. He has, like the physician, to possess a general knowledge of disease, and to possess, like the surgeon, a certain skill of hand; but besides this be has to acquire a special manual dexterity never called for in a surgeon, and to possess a special knowledge of metallurgy, of chemico-physics, and of branches of mechanics of which neither the physician nor the surgeon need know anything at all.

All knowledge is useful, but the power of the human mind to attain and retain knowledge is limited. We cannot all know everything. The surgeon need not, and, if he is to excel greatly in his art, cannot, know all the minutiæ of the physician's calling; he cannot at once be an accomplished surgeon and complete master of all the details of auscultation and the intricacies of neural pathology. In like manner the dentist, if he is to excel in his art, cannot hope to know all that the physician must know and the surgeon must know. Such being the case, where shall we begin to narrow the education of the dentist? for narrow it we must. How are we to differentiate the training of this special healer from the training of the general healer, the physician or the surgeon?

The training of the doctor is partly general, partly special. His special training ought to be as full and as complete as possible; he cannot know too much, he cannot be taught too much of actual disease and of the various means to combat it. His general training stands on a different footing. The object of this is to enable him to understand and judge the special knowledge which he has to acquire, and though from one point of view no general education can be too wide, from the point of view of the demands of actual life that general education is sufficient which secures the above object and which adequately prepares him for the special training which follows. The main elements of the doctor's general training are these: He must know general pathology, the nature of the processes of disease. This is the central element, the fundamental element, absolutely necessary for the understanding of the true nature of individual maladies, and time spent on this is time wisely and economically spent. Further, he must know physiology and anatomy, but there is no need to carry his studies in these farther than is sufficient to enable him clearly and fully to lay hold of the truths of pathology and the laws of health, and to impress on his mind such details of topographic anatomy as will always stand him in good stead in his practice as a physician or a surgeon. Lastly, he must know physics and chemistry, for without a certain knowledge of these he cannot understand physiology, and must remain really ignorant of pathology.

The dentist, like the doctor, needs a general as well as a special training. What can be said about the general education of the dentist? And when I say "dentist" I mean the scientific dentist, he who does his work not by mere rule of thumb, but in the light of scientific knowledge and under the guidance of scientific principles,—for it is with him alone, I take it, that we are interested here. What ought the scientific dentist to undergo in the way of general training?

I imagine that I shall not go far wrong when I say that, in common with the doctor, he ought to possess a general knowledge of pathology. He has to deal with disease, with disease of the teeth and, indeed, of the mouth, and he ought to be well acquainted with the general truths of pathology. He need not be carried farther into the details of disease than is necessary to enable him to understand general morbid processes and the common ways in which living structures go wrong. But he may with profit be led to spend some considerable time on that division of pathology which teaches how many of the ills that flesh, even the hardest part of it, is heir to are the handiwork of minutest organisms, are scourges laid on by invisible rods. What we now call bacteriology must, so far as it deals with disease, be an essential part of every dentist's training. Beyond this the dentist needs, like the doctor, such knowledge of physiology and anatomy as will enable him to lay hold securely of pathology, but in his case the details of the topographic anatomy of the body at large are not needed, and may fitly give place to a knowledge of the anatomy and physiology of the teeth, more special and more complete than is ever needed by any doctor. Such a general training is one more or less common to both the dentist and the doctor. But the former has also need of a general—that is, of a preparatory—training wholly uncalled for in the case of the latter.

The days when the public mainly judged of the merits of a dentist by the celerity and freedom from pain with which he robbed his patient of possessions which could never be really replaced are long gone by. The art of the dentist is now pre-eminently a constructive and preservative art. And the dentist, if he is to succeed in construction, must know the nature of the materials which he constructs, and the physical, mechanical laws of the construction which he attempts. If, in order to grapple adequately with disease, he must share in the general training of the doctor, he must, in order to grapple with the difficulties of repairing the ravages of disease which he and others have failed to prevent, share in another general training of a wholly different kind. He must be inducted into some, at least, of the mysteries of metallurgy; he must have a scientific knowledge of the chemical and physical properties of the varied materials which he uses for construction, and he must learn something of what may be described as a special branch of engineering. He must be trained in ways and things wholly unknown to the physician and the surgeon. Moreover, if he is to hope to succeed in his profession, he must know the things of which I am speaking not only theoretically, but practically. Just as the young doctor begins his practical hospital duties by dressing wounds and acting as a nurse, as the general who commands armies has at the beginning to take his place as a private in barrack square drill, as the young engineer puts on his blouse to go through the workshop, so the young dentist must spend an allotted time at the bench.

Obviously the training of the dentist, much as there must be in it common with that of the doctor, must be narrowed in its own way if the cone of education is to be brought to an effective apex.

Doubtless the dental profession has much to gain in many ways by a close alliance with the medical profession. The position of being a branch of the great and powerful medical profession gives it advantages many and great, and it would be folly to cast away these advantages by demanding a divorce unless that divorce be really necessary.

One object, and one object only, ought to be the aim of the training of a dentist,—to make him as sure and as efficient a workman as possible. If, as seems probable, in the rush of men and things, ordinary minds under ordinary circumstances cannot achieve that efficiency and at the same time pursue a complete medical education, then some separation seems inevitable. The separation, however, should not be a divorce, but simply a deviation or differentiation, a claim for a separate apex hand in hand with the acknowledgment of a common basis.—*Dental Cosmos.*

(To be continued.)

